# Tibial Condyle Valgus Osteotomy for Ipsilateral Knee Osteoarthritis after Hip Arthrodesis

**DOI:** 10.1155/2021/6443618

**Published:** 2021-10-29

**Authors:** Daisuke Fukuhara, Hiroaki Inoue, Shuji Nakagawa, Yuji Arai, Kenji Takahashi

**Affiliations:** ^1^Department of Orthopaedics, Graduate School of Medical Science, Kyoto Prefectural University of Medicine, 465, Kajiicho, Kawaramachi-Hirokoji, Kamigyo-Ku, Kyoto, Kyoto 602-8566, Japan; ^2^Department of Sports and Para-Sports Medicine, Graduate School of Medical Science, Kyoto Prefectural University of Medicine, 465, Kajiicho, Kawaramachi-Hirokoji, Kamigyo-Ku, Kyoto, Kyoto 602-8566, Japan

## Abstract

We report a case of tibial condylar valgus osteotomy (TCVO) for ipsilateral knee osteoarthritis (OA) after hip arthrodesis. A 58-year-old woman developed right purulent hip arthritis at one month of age and underwent right hip fusion at 16 years old. She visited our department at the age of 57 because her right knee joint pain worsened. The range of motion for her right knee was 80° and -5° of flexion and extension, respectively, and she experienced medial weight-bearing pain. A plain X-ray image showed that the right knee joint had end-stage knee OA with a bone defect inside the tibia, and the tibial plateau shape was the pagoda type. There was a marked instability in her right knee with a valgus of 9° and varus of 7° on stress photography. She underwent TCVO on her right knee and was allowed full load four weeks after surgery. Computed tomography imaging showed bone union nine months after surgery. Two years after the operation, there was no correction loss, and she could walk independently without pain. In general, total knee arthroplasty (TKA) is indicated for end-stage knee OA; however, there are problems, such as early loosening due to the increased mechanical load on the knee after hip OA. In this case, since a good course was obtained, TCVO is considered a treatment option for terminal knee OA after hip arthrodesis.

## 1. Introduction

Several approaches using artificial joints are employed for the surgical treatment of ipsilateral knee osteoarthritis (knee OA) after hip arthrodesis. These approaches include total hip arthroplasty (THA) alone, total knee arthroplasty (TKA) after THA, and TKA alone. However, the long-term outcomes of the use of artificial joints in treating knee OA after hip arthrodesis are unknown. Therefore, other treatments should be considered, especially for nonelderly patients.

Tibial condylar valgus osteotomy (TCVO) allows the preservation of the knee joint. This procedure improves knee joint instability by changing the shape of the proximal tibia so that both condyles can come into contact simultaneously [1, 2].

Osteotomies, such as TCVO, are generally indicated for advanced to terminal knee OA, and positive results have been obtained [1]. However, to the best of our knowledge, there are no reports of joint-sparing surgery for knee OA following hip fixation. We report a case in which the use of artificial joints was avoided through the selection of TCVO, with a positive postoperative course.

## 2. Report of the Case

The patient was a 58-year-old woman (height, 138 cm and weight, 39 kg). She developed right hip septic arthritis at the age of 1 month and underwent arthrodesis of the right hip at the age of 16 years. At 53 years, posterior lumbar interbody fusion was performed for degenerative spondylosis.

The patient first visited our department at age 57, with complaints of increasing pain in the right knee. Upon physical examination, we discovered muscular atrophy from her right hip to the lower limbs, with a reduction of approximately 7 cm in the difference of her thigh circumference (measured at 10 cm from the upper edge of the patella). Her right hip was fixed at a flexion of 25° and an abduction of 10°. The range of motion in her right knee was 80° of flexion and −5° of extension, with medial weight-bearing pain. Her right leg was 2 cm shorter than her left leg in spinamalleolar distance, and we observed patellar ballottement in her right knee. She also experienced difficulty in daily life activities.

**Figure 1:**
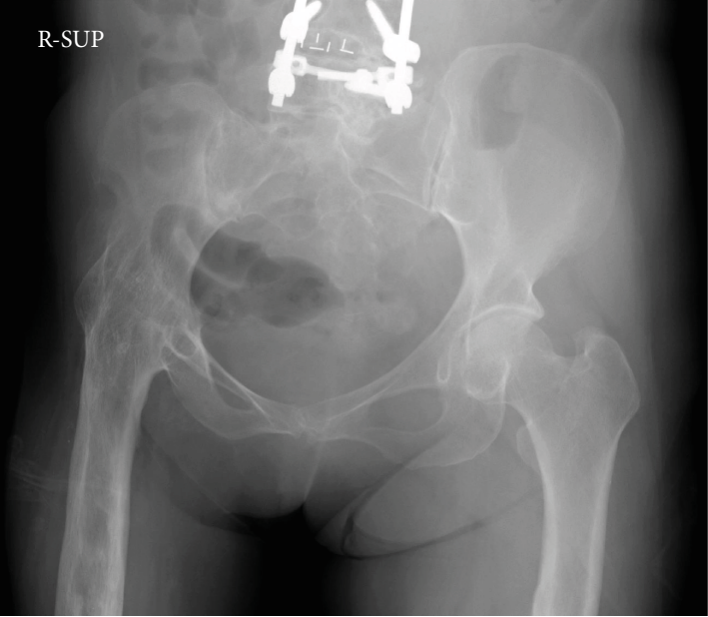
Radiography of the pelvis shows arthrodesis of the right hip joint and posterior lumbar interbody fusion.

**Figure 2:**
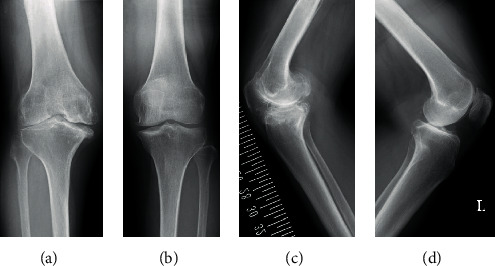
Radiography shows grade 4 osteoarthritis of the right knee joint, according to the Kellgren-Lawrence scoring system, with a pagoda-type tibial plateau. (Right (a) and left (b) anterolateral view and right (c) and left (d) lateral view).

**Figure 3:**
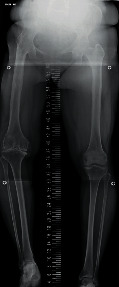
Full-length radiography of the lower limbs shows pelvic tilt and right genu varum.

**Figure 4:**
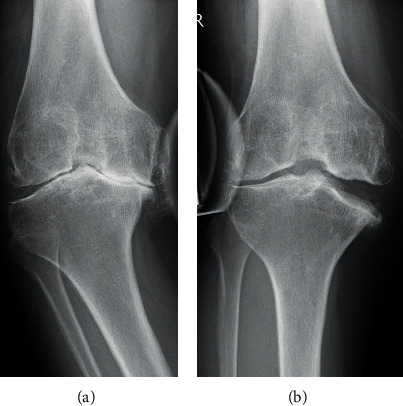
Stress radiography shows remarkable instability of the right knee. (Varus (a) and valgus (b)).

**Figure 5:**
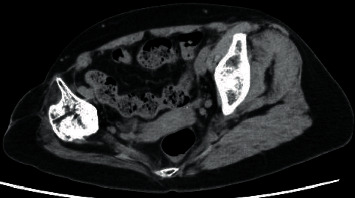
Computed tomography of the hip joint shows marked atrophy of the right gluteus medius muscle.

The patient's Japanese Orthopaedic Association (JOA) scores for knee OA were 55 of 100 points, whereas her knee scores were 65 of 100 points on the affected side (right side). Using radiography, we observed the fusion of the right hip joint at an abduction of 10° (Figure 1) and determined that the extent of OA on the healthy side (left side) was slight. The image of the right knee joint revealed grade 4 OA on the Kellgren-Lawrence scoring system, with a bone defect in the medial tibia. The shape of the tibial plateau was a pagoda-type, and the medial tibial plateau depression (MTPD) [3] was −40° (Figure 2). Full-length radiography of the lower limbs showed pelvic tilt and genu varum, with a femorotibial angle of 182° (Figure 3). It was impossible to obtain the single-legged stance view of the affected limb due to severe pain and instability. Significant instability of the knee was also observed using stress imaging, with valgus and varus instabilities of 9° and 7°, respectively (Figure 4). Computed tomography (CT) of the hip joint demonstrated marked atrophy of the right gluteus medius muscle (Figure 5). Blood examinations showed that C-reactive protein level, erythrocyte sedimentation rate, and white blood cell count were within their normal ranges, and no findings suggested infection.

**Figure 6:**
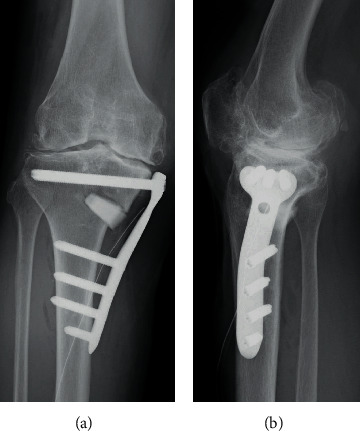
Postoperative radiography shows that tibial condyle valgus osteotomy with an open distance of 11 mm was performed and fixed with a metal plate. (Anteroposterior view (a) and lateral view (b)).

**Figure 7:**
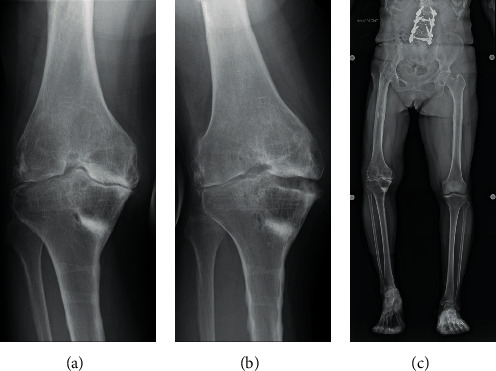
Knee instability improved after the removal of the metal implants (varus (a) and valgus (b)), and a double-legged stance view shows no correction loss at 2 years and 9 months postoperatively (c).

Based on the above, the patient was diagnosed with severe right knee OA following right hip arthrodesis and she underwent surgery. Using the International Cartilage Research Society classification, which indicates cartilage damage using arthroscopic findings, we identified grade 2 damage on the patella side of the patellofemoral joint and grade 4 damage on the femoral side. Grade 4 damage was determined in all compartments of the femorotibial joint. In accordance with the technique described by Chiba et al. [1], TCVO, with an open distance of 11 mm, was performed and fixed using the Olympus TriS Medial HTO plate system standard type (Olympus Terumo Biomaterials, Tokyo, Japan). The open distance was determined by opening the osteotomy site until contacting the femoral and tibial medial condyle. The postoperative outcome was positive. The patient's MTPD improved to −30° (Figure 6), and she attained full range of motion in her right knee immediately after surgery. The patient began with partial weight bearing immediately after surgery, and full weight bearing was achieved 4 weeks subsequently. A CT image taken 9 months after the operation revealed bone union of the right tibia. The metal plate was removed 13 months postoperatively. At the final follow-up 2 years and 9 months after the operation, no correction loss was observed and the patient could walk independently, with no pain. The right knee joint displayed a flexion of 70° and an extension of −5°, and stress radiography showed a valgus instability of 5° and a varus instability of 3°, demonstrating improved stability in the right knee and better walking ability (Figure 7). Her JOA scores for knee OA and knee scores at the final follow-up were 65 and 64 points, respectively.

## 3. Discussion

Hip arthrodesis connects the pelvis and femur, resulting in the loss of hip mobility. In addition, shortening of the affected limb causes a difference in leg length, which increases the mechanical load applied to the adjacent joint. This may result in subsequent spondylosis of the lumbar spine and/or arthritic changes in joints, such as the knee and sacroiliac joints. Notably, the incidence of knee OA following hip arthrodesis is approximately 65% [4, 5]. Both varus and valgus knee deformities have been reported; however, joint instability due to mediolateral ligament imbalance is likely to occur regardless of the alignment. Furthermore, the patient's walking ability is often extremely reduced due to pain and a limited range of motion in the affected knee joint.

In our case, the lumbar spine and the knee joint adjacent to the fused hip joint were damaged. Several treatment options were available. In a previous report, when THA was performed on a fused hip joint, the improvement rate of knee pain ranged from 20% to 91% [6]. Rittmeister et al. also described a positive postoperative course when THA was combined with TKA [7]. However, the application of THA after hip arthrodesis often causes residual limping and a feeling of instability in the hip joint due to atrophy of the gluteus medius muscle. Furthermore, there is a higher risk of dislocation and nerve damage than in standard THA [8, 9]. In addition, if infection was the reason for the arthrodesis, as in this case, THA would be associated with the risk of recurrent infection [10]. Therefore, the indication for THA after hip fixation should be carefully determined. In this case, we considered the risk to be too high to complete THA, since CT revealed severe atrophy of the gluteus medius muscle and she experienced septic hip arthritis in early childhood.

Romness et al. reported that knee pain after hip arthrodesis improved when only TKA was performed for ipsilateral knee OA in a good limb position [11]. Generally, TKA greatly reduces the gonalgia experienced with knee OA, but several problems may be encountered after hip arthrodesis. First, because the knee joint is placed in a specific mechanical environment, early failure of TKA may occur. Next, during TKA, the femoral component is positioned to obtain a load plane perpendicular to the functional axis of the lower limbs on the coronal plane. However, the pelvis and femur are connected after hip arthrodesis, making it difficult to devise an appropriate preoperative plan. In addition, it is necessary to predetermine the intraoperative position, and setting the implant was more difficult than was expected [12, 13].

Considering these problems and the patient's age, in this case, we discussed the possibility of joint-preserving surgery. Joint-preserving surgeries for genu varum include closing wedge high tibial osteotomy (CWHTO), open wedge HTO (OWHTO), and TCVO. These procedures aim to correct the alignment of the lower limbs and change the load environment around the knee joint, mainly by correcting the tibia [14]. This case had no indication for extra-articular correction, as seen with CWHTO and OWHTO, because of the intra-articular deformity (the patient's MTPD was −40°). In addition, devising an appropriate preoperative plan for OWHTO and CWHTO is difficult, because evaluation of the functional axis of the lower limb is not performed as comprehensively as in TKA.

Conversely, TCVO, which enables joint preservation, reconstructs the tibial joint surface by osteomizing and lifting the medial condyle of the tibia. This technique is well indicated for genu varum with a pagoda-type intra-articular deformity. TCVO aims to improve knee joint symptoms caused by varus−valgus instability, by emphasizing the compatibility of the proximal tibial joint surface rather than the functional axis of the lower limbs [1]. Therefore, we considered that TCVO would improve the symptoms of ipsilateral knee OA, with significant instability, after hip arthrodesis.

Furthermore, joint laxity is also associated with the pathophysiology and progression of knee OA [15]. TCVO not only improves knee instability; it may also prevent the progression of knee OA. Chiba et al. have reported that TCVO improved the knee instability and resulted in the good clinical outcome for at least 5 years [1]. In this case, the patient's MTPD improved from −40° preoperatively to −30° postoperatively, and her varus−valgus instability also improved from 16° preoperatively to 8° postoperatively. Two years and nine months after the operation, she experienced no feelings of instability or pain during walking, and a positive postoperative course was obtained. TCVO has inhibited the progression of knee OA in this case although it was short-term. Long-term follow-up is required for the knee OA inhibitory effect.

Caution is required in indicating the use of prosthesis for adolescent patients. TCVO is a viable treatment option for nonelderly individuals with unstable knee OA after ipsilateral hip arthrodesis.

## Data Availability

No data were used to support this study.
